# Stress urinary incontinence: Sling or colposuspension?

**Published:** 2007

**Authors:** Rajiv Paul Mukha, J. Chandra Singh, Nitin S. Kekre

**Affiliations:** Department of Urology, Christian Medical College, Vellore, India. E-mail: chandrasingh@cmcvellore.ac.in

## SUMMARY

This study was a multicenter, randomized clinical trial comparing pubovaginal sling using autologous rectus fascia and Burch colposuspension among women with stress urinary incontinence. Women were eligible for the study if they had predominant symptoms of stress urinary incontinence, a positive stress test and urethral hypermobility. The primary outcomes were success in terms of overall urinary incontinence measures which required a negative pad test, no urinary incontinence (as recorded in a three-day diary), a negative cough and Valsalva stress test, no self-reported symptoms and no retreatment for the condition. Postoperative urge incontinence, voiding dysfunction and other adverse events were also assessed. Six hundred and fifty-five women were randomly assigned to two study groups to undergo either the sling procedure (326) or Burch colposuspension (329). Five hundred and twenty women (79%) completed the outcome assessment. At 24 months, success rates were higher for women who underwent the sling procedure than for those who underwent the Burch procedure, for both overall category of success (47% vs. 38%, *P* = 0.01) and the category specific to stress incontinence (66% vs. 49%, *P*<0.001). However, more women who underwent the sling procedure had urinary tract infections, difficulty in voiding and postoperative urge incontinence. Hence the authors have concluded that autologous fascial sling results in a higher rate of successful treatment of stress incontinence than Burch colposuspension but the morbidity with the sling was higher than with colposuspension.

## COMMENTS

This study[1] compares the outcome following autologous fascial sling versus Burch colposuspension for stress urinary incontinence. Incontinence rates as high as 69% have been reported in the community.[2] The ideal manner with which to report the outcomes of surgical interventions—or for that manner any intervention for any disease or symptom state—remains unsettled. This issue is particular for all trials; however, for a study affecting quality of life, this is more significant.[3] Even in multicentric trials, interpretation of results may be difficult if the selection criteria, measures of efficacy and frequency of follow-up are not adequate.[4] This well-conducted prospective, randomized multicentric trial compared autologous fascial sling with Burch colposuspension for stress incontinence. The subjects were randomized just prior to surgery leaving out room for bias on the part of the surgeon. The selection criteria were fairly uniform. The two procedures were standardized and the two main outcomes were composite measures of success in terms of overall continence and stress incontinence specifically. Both Burch colposuspension and the sling procedure have been reported to have success rates of 70-80% at five to eight years.[5,6] In this study, though the initial cure rates were high, there was a decline in the cure rates over time emphasizing the need for a longer study period. The higher success rates in the sling group were partly offset by a higher number of urinary tract infections, urge incontinence, voiding dysfunction and a greater number of reoperations to improve voiding. An important fact highlighted by the editorial[7] on this article was that if success rates were measured based purely on the satisfaction of the women, success rates would be even lower. To a surgeon, an occasional leak may be acceptable but to a woman who works in a busy corporate office or is a college lecturer it may be a social embarrassment. Also, the benefit of these procedures is partly offset by the onset of symptoms of urgency and urge incontinence. Similar to autologous fascial sling, TVT when compared to Burch colposuspension was found to have better results.[8] In conclusion, this is a well-conducted clinical trial with adequate sample size to compare two established operative procedures. The autologous fascial sling has a better outcome in terms of cure rates. Erosion of slings, which is more relevant to synthetic slings, is one aspect that has not been studied. This study will be helpful in counseling patients as to a procedure for stress incontinence. However, trials with a longer follow-up and addressing patient satisfaction primarily will be more useful to assess the impact on the patient's quality of life.
